# Implementing goals of care conversations with veterans in VA long-term care setting: a mixed methods protocol

**DOI:** 10.1186/s13012-016-0497-0

**Published:** 2016-09-29

**Authors:** Anne E. Sales, Mary Ersek, Orna K. Intrator, Cari Levy, Joan G. Carpenter, Robert Hogikyan, Helen C. Kales, Zach Landis-Lewis, Tobie Olsan, Susan C. Miller, Marcos Montagnini, Vyjeyanthi S. Periyakoil, Sheri Reder

**Affiliations:** 1 Center for Clinical Management Research, VA Ann Arbor Healthcare System, Ann Arbor, MI USA; 2 University of Michigan Medical School, 300 N. Ingalls Street, Room 1161-I, Ann Arbor, MI 48109-5423 USA; 3 Corporal Michael J. Crescenz VAMC, Philadelphia, PA USA; 4 School of Nursing, University of Pennsylvania, Philadelphia, PA USA; 5 Canandaigua VAMC, Canandaigua, NY USA; 6 University of Rochester Medical Center, Rochester, NY USA; 7 Eastern Colorado Health Care System, Denver, CO USA; 8 School of Medicine, University of Colorado Anschutz Campus, Denver, CO USA; 9 Brown University School of Public Health, Providence, RI USA; 10 VA Palo Alto Health Care System, Palo Alto, CA USA; 11 Stanford University School of Medicine, Stanford University, Palo Alto, CA USA; 12 Puget Sound Health Care System, Seattle, WA USA

**Keywords:** Long-term care, Goals of care conversations, Implementation science, Feedback interventions, Learning collaboratives

## Abstract

**Background:**

The program “Implementing Goals of Care Conversations with Veterans in VA LTC Settings” is proposed in partnership with the US Veterans Health Administration (VA) National Center for Ethics in Health Care and the Geriatrics and Extended Care Program Offices, together with the VA Office of Nursing Services. The three projects in this program are designed to support a new system-wide mandate requiring providers to conduct and systematically record conversations with veterans about their preferences for care, particularly life-sustaining treatments. These treatments include cardiac resuscitation, mechanical ventilation, and other forms of life support. However, veteran preferences for care go beyond whether or not they receive life-sustaining treatments to include issues such as whether or not they want to be hospitalized if they are acutely ill, and what kinds of comfort care they would like to receive.

**Methods:**

Three projects, all focused on improving the provision of veteran-centered care, are proposed. The projects will be conducted in Community Living Centers (VA-owned nursing homes) and VA Home-Based Primary Care programs in five regional networks in the Veterans Health Administration. In all the projects, we will use data from context and barrier and facilitator assessments to design feedback reports for staff to help them understand how well they are meeting the requirement to have conversations with veterans about their preferences and to document them appropriately. We will also use learning collaboratives—meetings in which staff teams come together and problem-solve issues they encounter in how to get veterans’ preferences expressed and documented, and acted on—to support action planning to improve performance.

**Discussion:**

We will use data over time to track implementation success, measured as the proportions of veterans in Community Living Centers (CLCs) and Home-Based Primary Care (HBPC) who have a documented goals of care conversation soon after admission. We will work with our operational partners to spread approaches that work throughout the Veterans Health Administration.

**Electronic supplementary material:**

The online version of this article (doi:10.1186/s13012-016-0497-0) contains supplementary material, which is available to authorized users.

## Background

Goals of care (GoC) conversations are critical in long-term care settings. Older people in these settings are vulnerable, frail, and often face critical decisions about future care, particularly life-sustaining treatments (LSTs). Many have experienced serious health problems that could trigger the need for LSTs, or are likely to experience such within the next year. Conversations about LST preferences are frequently held with veterans and their families, and documentation of care preferences in an advance directive is routine in most Veterans Health Administration (VA) settings. While admission to a long-term care service or setting is an appropriate trigger for GoC conversations, practices vary across VA Community Living Centers (CLCs) and VA Home-Based Primary Care (HBPC) programs about how and when these conversations should be held after admission.

The VA National Center for Ethics in Health Care has updated Handbook 1004.03, “*Life Sustaining Treatment Decisions: Eliciting, Documenting, and Honoring Patients’ Values, Goals, and Preferences*”. Through this update, licensed prescribing practitioners throughout all care delivery settings in the VA will be required to hold GoC conversations with veterans and their families, document the findings in a standard template installed in a consistent, prominent place within the VA electronic health record, and develop care plans consistent with the goals expressed by the veterans and their families. While the focus of the conversation is on LST decisions faced by veterans with life-limiting events (such as end-stage heart failure or terminal cancer), the intent is to elicit, document, and respect veterans’ preferences for care, which is important for all veteran populations.

The evidence for the VA LST initiative and similar programs is mixed [1], but a recent narrative review describes improved outcomes for patients who have engaged with their clinicians in GoC discussions [2]. Outcomes that may be improved by timely GoC include better quality of life for patients, less aggressive medical care near the end of life, and earlier referral to hospice. Family members report better adjustment to bereavement [2]. Studies have also shown that these improved outcomes can be achieved without increasing anxiety, depression, and hopelessness for patients, and can lead to a reduction in distress for surrogates who make decisions about end-of-life care for family members [2]. Our primary rationale for engaging in this program of implementation support is the fact that the GOC conversation program has been initiated by VA’s national program office, with which we are partnering.

The new requirements for timing and documentation of GoC conversations provide a unique opportunity for CLC and HBPC staff to improve current practices by ensuring that these conversations take place and are documented using the new standardized template specified in the updated handbook. Implementation of complex interventions such as GoC conversations, which require significant and sustained behavior change on the part of clinical team members, veterans, and their families, is a process that takes time and focused attention to ensure it is sustained and becomes the norm, a “culture change”. A shared decision-making framework can support this effort by providing veterans and their families or caregivers with information and tools to more fully participate in the process. Our team for the Quality Enhancement Research Initiative (QUERI) program, which includes an Implementation and Data Core, will test the design and delivery of implementation strategies to optimize integration of the LST across several VA regional networks and facilities. Our overall goal is to support veteran-centered implementation of GoC conversations across the long-term care services and supports continuum in the VA. This program will engage a diverse group of investigators and VA operational partners, including the National Center for Ethics in Health Care, Geriatrics and Extended Care program offices, and the Office of Nursing Services.

We will accomplish our overall impact goal through the following specific aims:

1. Assess variation in practice measures related to implementation of GoC conversations in CLCs and HBPC programs over time nationally
2. Design, implement, and test tools to improve performance in five geographically diverse VA regional networks, specifically:
  - Feedback reports to provide information on progress towards key processes and outcomes
  - Learning collaboratives to support action planning to address performance gaps
3. Use measures derived from two widely used implementation research frameworks, the Consolidated Framework for Implementation Research and the Theoretical Domains Framework to assess barriers and facilitators to implementing GoC conversations
4. Use audit with feedback interventions, coupled with action planning through learning collaboratives to conduct rapid tests of different designs within these strategies to overcome barriers to implementing GoC conversations in CLCs and HBPC

## Methods/Design

### Overview of the three program projects

We propose two projects focused on implementing GoC conversations in CLCs, which are institutional settings, and one in HBPC environments that extends our implementation efforts to non-institutional settings. These projects will be supported by our Implementation and Data Core (IDC) in deploying our common implementation strategy, audit with feedback coupled with action plans developed through learning collaboratives. The proposed IDC will collect and report process and outcome measures using routinely collected administrative and clinical data combined with data collected as part of the implementation processes. We note that in all projects, and throughout the Program, as we discuss eliciting GoC from veterans, we will also involve family members or other surrogates as necessary, depending on an individual veteran’s cognitive status and ability to participate.

Our work is planned as quality improvement (QI). The VA has developed policies which permit operational QI projects to be published under oversight from local, regional, or national program offices (http://www.va.gov/vhapublications/searchresults.cfm?ST=Adv&RPP=20&SQ=www.va.gov/vhapublications&RS=741). In this case, our program has been deemed QI by the VA Ann Arbor Healthcare System Research and Development Committee, and is being conducted under the auspices both of national program offices (the VA National Center for Ethics in Health Care and Geriatrics and Extended Care) and local programs (local and regional Geriatrics and Extended Care committees).

The first of the three projects, *Implementing Goals of Care Conversations in CLCs*, is intended to be completed in the first year of the 5-year program. The goal is to work closely with CLCs in one regional network to implement the guidance in the updated Handbook, focusing on supporting the implementation of eliciting goals related to life-sustaining treatment and preferences regarding the setting of care.

The second project, *Partnership to Enhance Resident Outcomes: Collaborative Care Plans for CLC Residents with Dementia*, will be conducted during years 2 through 5. It also extends the Handbook guidance and builds on GoC conversations and documentation of veteran preferences to develop personalized care plans for veterans with dementia living in CLCs in two regional networks. Project 2 focuses on deepening the care team’s use of goals, preferences, and values elicited during the GoC conversations by expanding discussions to encompass choices about therapeutic approaches for pain and other symptoms; use of medications and non-pharmacologic strategies for behavior management; and honoring lifestyle preferences (for example, waking, meals, sleeping times, and dietary choices) that affect quality of life.

The third project, *Implementing Goals of Care Conversations in HBPC*, will be conducted during years 2 through 5 of the program. Similar to the first project, the overall goal is to support implementation of the updated Handbook guidance in HBPC programs in two additional regional networks. As in the first project, we will support staff in completing and documenting GoC conversations during the first or second HBPC visit.

Further information about the first and third projects are given in Additional file 1. Project 2 will be described in a separate manuscript. We will form a Steering Committee comprised of our operational partners, a family member of a veteran who died in a VA CLC, leadership from our Program, and others as recommended by the Quality Enhancement Research Initiative (QUERI) Program, which is our primary funding body.

### Implementation and Data Core

The Implementation and Data Core (IDC) will work in concert with the full project team to support our aims. The data component of the Core will compile, analyze, and report both routinely collected administrative and clinical data (described in Table 1) from several sources within VA, as well as data collected specifically for this project. The IDC will employ strategies to support integration of best practices and minimize variation in practices across sites. In all cases, a key underlying principle is to keep the implementation burden on staff to a minimum, by supporting project efforts through the IDC to the greatest extent possible.

**Table 1:** Program outcomes and measures

Data element	Source	Feedback reports	Process evaluation	Project(s)
Data from life-sustaining treatments (LST) template				
Veteran newly admitted to CLC or HBPC^a^	CDW Inpatient	Yes	No	1,2,3
LST template completed in 7 days^a^ in CLC or by the second visit in HBPC	CDW Health Factors	Yes	No	1,2,3
Proportion veterans endorsing full LST^b^	CDW Health Factors	Yes	Yes	1,3
Proportion veterans endorsing other than full LST^b^	CDW Health Factors	Yes	Yes	1,3
Proportion veterans expressing understanding of medical facts^b^	CDW Health Factors	Yes	Yes	1,3
PROMISE Center Data				
Q18 from Bereaved Family Survey: Overall, how would you rate the care he/she received in the last month of life?^a^	Bereaved Family Survey	No	Yes	1,3
Minimum Data Set (MDS) 3.0 Nursing Home Resident Assessment Data				
Veteran preferences for customary routine and activities^b^	MDS 3.0 Section F	No	Yes	1
Mood (PHQ-9)^b^	MDS Section D	No	No	1,2,3
Behavioral symptoms^b^	MDS 3.0 Section E	No	No	1,2,3
Pain^b^	MDS 3.0 Section J	No	No	1,2,3
Dyspnea^b^	MDS 3.0 Section J	No	No	1,2,3
Falls^b^	MDS 3.0 Section J	No	No	1,2,3
Medications: antipsychotics, anxiolytics, mood stabilizers, analgesics^b^	MDS 3.0 Section N	No	No	1,2,3
VA administrative data				
Healthcare utilization variables, hospitalizations, ER visits^b^	CDW, VA Decision Support System, Vital Status	Yes	No	1,2,3
Medications: antipsychotics, anxiolytics, mood stabilizers, analgesics^b^	VA Decision Support System	Yes	Yes	2
Burdensome transitions^b^	RHF (incorporating VA and CMS data)	Yes	Yes	1,2,3
End-of-life quality indicators^b^	BFS, GEC DAC	Yes	Yes	1,2,3
Leadership and unit culture^c^	All Employee Survey	No	Yes	1,2,3
Artifacts of Culture Change tool				
Leadership and Outcomes domains^c^	Artifacts of Culture Change tool	No	Yes	1,2
Data collected as part of the program				
ORCA evidence scales^c^	ORCA survey	No	Yes	1,2,3
ORCA context scales^c^	ORCA survey	No	Yes	1,2,3
Barrier assessment^c^	CFIR and TDF	No	Yes	1,2,3
Feedback uptake scale^c^	Feedback uptake survey	Sent shortly after feedback report sent	Yes	1,2,3
Action plan completion^c^	Interviews	No	Yes	1,2,3

Key (how variables are used in analysis):

^a^Primary outcome

^b^Secondary outcome

^c^Covariate

#### Common implementation strategies

We have specifically selected a coupled strategy of audit with feedback and learning collaboratives to generate action planning in order to optimize the effect of both strategies. Both strategies have been used extensively and been found to be associated with mixed effectiveness in promoting desired provider behavior change [3–8]. Each has been studied, although with differing levels of rigor. Although there is considerable information about some aspects of each strategy, and despite the fact that learning collaboratives can provide action planning, required for optimal feedback uptake, they have not been studied as a coupled intervention [9]. Further detail on our implementation strategies is provided in Additional file 2.

### Implementation activities

#### Guiding frameworks

Our program is guided by two implementation frameworks: Consolidated Framework for Implementation Research (CFIR) and the Theoretical Domains Framework (TDF). Our primary rationale for using both frameworks is that one (TDF) specializes in individual-level behavior change, while the other (CFIR) focuses more on the organizational level, above the individual. The CFIR is a widely used framework in implementation research [10]. It has a number of linked interview guides, and a question repository stored in the CFIR Wiki (www.cfirguide.org). It was initially developed by VA investigators and has been adopted by many VA and non-VA groups in the USA and other countries. It has primarily been applied through semi-structured individual and focus group interview guides to elicit barriers and facilitators in five domains: Intervention Characteristics; Outer Setting; Inner Setting; Characteristics of Individuals; and Process. Survey instruments based on the CFIR have been developed and are currently under review.

The TDF is also a widely used framework in implementation research that was initially developed from health behavior change theories focused primarily on the individual level [11]. It has since been used in many implementation studies [12], and several interview guides have been developed. Like the CFIR, it has primarily been used qualitatively, administered through semi-structured interviews; however, a survey instrument has been developed [13] based on the most current version of the TDF [14]. This updated version has 14 TDF domains: Knowledge; Skills; Social/Professional Role and Identity; Beliefs about Capabilities; Optimism; Beliefs about Consequences; Reinforcement; Intentions; Goals; Memory, Attention and Decision Processes; Environmental Context and Resources; Social Influences; Emotions; and Behavioral Regulation. Ongoing research is developing the ability to link identified barriers to *implementation strategies* and *behavior change techniques* [15, 16]. These are important tools for implementing change at both the individual and organizational/policy levels.

*Implementation strategies* are described [17–19] as theoretical and/or empirically tested approaches that promote implementation of evidence-based practices. These strategies, a total of 73 described in this body of work, range from “Access new funding” to “Work with educational institutions” [19]. They function at multiple levels, from strategies focused on individuals to those focused on policy and organizational change. In many cases, these strategies are “umbrella” approaches, within which there is still a need to design and specify content to create implementation interventions. This idea was described in a paper published in 2006, which was derived from early QUERI efforts to change behavior related to a number of clinical areas [20]. Linkages between CFIR constructs identified as barriers or facilitators and implementation strategies are being developed.

*Behavior change techniques* have been collated and described extensively in work coming from a multidisciplinary group in the UK that developed the TDF. In this body of work, 93 different behavior change techniques are clustered hierarchically in a Behavior Change Techniques Taxonomy [16]. These techniques can be linked to constructs within TDF domains, which will allow us to design elements into the feedback and learning collaborative strategies. We regard the behavior change techniques as “atomic” units that can provide highly specific components to implementation interventions, and can be used to design elements within broader strategies. Using both the “umbrellas” of feedback and learning collaborative implementation strategies and the “atomic” units of individual behavior change techniques, we will test different design elements within these two strategies. We will also be able to tailor these strategies to local contexts through the data we gather from the context and barrier/facilitator assessments.

#### Assessing context and barriers and facilitators to implementing GoC conversations

We will use the Organizational Readiness to Change Assessment (ORCA) survey instrument [21] together with interviews and focus groups conducted using the CFIR [10] and the TDF [11] to obtain a multi-faceted view of the context of each unit in which we will be working (CLC unit, HBPC team). As we progress through the program, we will develop an expanding database of information about local context in CLCs and HBPC teams throughout the participating VA regional networks. We will use this information to tailor our interventions to accommodate variations in individual settings for maximal effectiveness.

The ORCA survey consists of three domains: Evidence, Context, and Facilitation. We will use the evidence and context domains, as these are relevant to an initial assessment. These two domains consist of 34 items, and the survey can be administered either in person using a pen and paper version or online. The ORCA is a validated [21] tool for assessing organizational readiness to change, an important factor in effective implementation [22]. In particular, organizational readiness to change is a construct in the CFIR, with important implications for intervention design. The IDC will collect all ORCA data, linked by role of the respondent and units/teams with which we are working.

For each project, we will use the data from unit-specific context and barrier/facilitator assessments to design tailored versions of our common implementation strategy described below. By doing this, we will build a database of information to support this kind of tailored implementation intervention design. For example, if we learn that providers are highly motivated to meet facility benchmarks for documented GoC conversations, we will add some form of comparison or benchmark to the feedback report to ensure that they have a comparison. Tailoring learning collaboratives might include providing scripts to support initiating GoC conversations if perceived self-efficacy in conducting them is low.

In addition to the use of contextual data for tailoring interventions, we will be able to develop variables for use as covariates in analyses of the effect of implementation interventions in specific settings. This latter use will allow us to compare processes, such as length of time to GoC completion after admission, and outcomes, such as repeat hospitalizations.

#### Contextualized experimentation using our implementation strategies

Small rapid cycle experiments, referred to as tests of change, are often used in QI work, and the strategies we are planning to use are common QI tools. Almost all QI projects include some form of performance feedback, and many use learning collaboratives. The fact that little is known about optimal design of these strategies affects the optimization of quality improvement, an activity that hospitals and healthcare systems across the globe use with regularity and on which they expend considerable resources in time, human energy, and money. Understanding how best to design and deploy these resources and tools is a critical business issue for all healthcare systems, while at the same time is of interest to researchers.

We are interested in supporting behavior change at the level of individual practitioner, initially, in having GoC conversations with new residents of CLCs or veterans newly admitted to HBPC programs. Providing feedback reports to these providers and the interdisciplinary teams working with them is expected to increase motivation to close performance gaps. Taking the further step of translating the elicited goals and preferences into care plans and from there into specific care activities will require behavior change on the part of the entire care team, including patients and families as feasible.

Learning collaboratives are one approach to team building, sharing among teams from different units so that shared learning can occur, and action plans can be developed to support closing performance gaps that appear in ongoing and changing feedback reports. As Weiner et al. notes, feedback reports require careful design to promote desired behavior change, as do learning collaboratives [23, 24]. We will use information from ongoing interviews and focus groups with staff in CLCs and HBPC teams to understand how different design elements work, and we will use individual behavior change techniques [16] intentionally designed into both feedback interventions and learning collaboratives as implementation strategies [19] to understand the effect of different design elements.

There are important questions about both audit with feedback interventions and learning collaboratives which we plan to address including:

- What is the optimal length of a feedback report designed to be delivered to individuals or groups working in healthcare settings? Does optimal length vary by role—whether feedback recipients are managers or frontline providers?
- What is the optimal format of feedback reports?
- What is the best way to deliver feedback for a given context?
- What information and tools are needed to support effective action plans by teams of providers?
- What are the most effective strategies for planning and operationalizing learning collaboratives to improve performance?
- How best to support action planning that includes goal setting?
- How best to use action plans developed through a learning collaborative to improve quality of care?

We plan to address these questions through brief, rapid quality improvement approaches that take advantage of the settings in which we will be working. In each project, we will conduct at least one experiment focused on either feedback reports or on learning collaboratives, which will be conducted as part of the work of that project. For example, we may vary mode of delivery of feedback reports across sites to assess the effectiveness of delivery through direct email vs. delivery through a supervisor. As a result of these QI experiments, we will generate useful knowledge about optimal approaches to using these strategies in combination. We will use the first and third projects, which focus on core implementation of the LST Handbook guidance, as the primary vehicles for this experimentation.

#### The data component of the Implementation and Data Core

The data component will use resources developed and maintained by VA’s Geriatrics and Extended Care Data and Analyses Center (GEC DAC), as well as those generated through the projects. The GEC DAC provides an infrastructure for acquisition and analysis of data from multiple VA sources to inform decision-making regarding the delivery, quality, and cost of geriatric and extended care programs, and is an integral data resource to the national VA Geriatric and Extended Care Offices for shaping policy and operations. Dr. Intrator’s work on the Residential History File [25] is foundational to the work we propose. The residential history file links Medicare and resident assessment data with VA administrative data to track care over time and in particular care provided in nursing homes. Through the GEC DAC, a VA/Medicare residential history file has been created that integrates data from VA’s utilization and costs databases with Medicare and Medicaid utilization and costs data to enable tracking of a person’s healthcare utilization over a variety of institutional and community-based healthcare settings, including, for example, HBPC. Importantly, the residential history file allows tracking of hospice use paid for by VA or Medicare, identifying whether it is provided in institutions and in the community. We will use the residential history file and other GEC DAC resources as sources of important variables. We show the full list of data sources and their planned use in this study in Table 1.

#### Data resource descriptions

The Bereaved Family Survey (BFS) is a 19-item tool to measure the quality and outcomes of end-of-life care. This nationwide survey is administered to the veteran’s next-of-kin shortly after their death in a VA inpatient (i.e., acute care, intensive care, or CLC) unit. The BFS is currently being piloted in several HBPC programs in three VA regional networks. The BFS is a valid tool that is endorsed by the National Quality Forum [26–29]. The VA has adopted one item (rating of overall care in the last 30 days of life) as a national performance measure. The facility-level BFS performance measure score reflects the percentage of respondents that rate care as “excellent.”

The MDS 3.0 is a comprehensive, standardized, federally-mandated assessment tool that must be completed for every nursing home resident, including those in VA CLCs at admission, discharge, and at regular intervals [30, 31]. The instrument measures many clinical outcomes that are critical in determining the needs, quality of care, and outcomes of nursing home residents. The MDS Version 3.0 has been extensively evaluated and the tool and its related indices that measure specific factors (e.g., mood) were found to be valid and reliable [30, 32].

Burdensome transitions are a concept developed by Gozalo et al. [33], to measure the degree to which nursing home residents experience care transitions near the end of life, with particular attention to transitions to acute care settings. The Residential History File provides comprehensive linked data to capture burdensome transitions as well as other quality indicators for end-of-life care. For the quality indicators of end-of-life care, we will adapt the indicators of poor end-of-life care identified by Earle and colleagues [34] and used by Gonsalves [35]. Indicators of poor end-of-life care include a hospice stay of less than 3 days, and one or more of the following in the last 30 days of life: ICU (intensive care unit) admission; two or more hospital admissions; hospital stay of 14 days or more; two or more emergency room visits; and non-hospice care transitions in the last 3 days of life.

The Artifacts of Culture Change (ACC) tool was developed by the Centers for Medicare and Medicaid Services (CMS) to measure the extent to which facilities have implemented 79 elements that reflect person-centered care [36]. The items are organized by six domains: Care Practices, Environment, Family and Community, Leadership, Workplace Practice, and Outcomes. Points assigned to each artifact in a domain are summed, yielding domain scores. Since FY 2007, the VA GEC office has required CLCs to submit data from the ACC (adapted for the VA) twice a year. Sullivan et al. [37] examined the association between ACC scores and a composite measure of quality (measured by the MDS 3.0) in 107 CLCs, and found significant associations, lending support for the positive influence of the culture change model on resident outcomes.

#### Analytic approaches to evaluation

We will use a primary strategy of interrupted time series/segmented regression analysis [38, 39] with matched comparisons to evaluate the impact of our implementation strategies. Matched comparison will be identified among CLC units for CLC evaluations and HBPC programs for HBPC evaluations. Veterans will be identified in CLC units based on the VA’ DSS WARD file which is derived from nurse staffing assignment to CLC units as related to particular Veterans in the CLC. Veterans cared for within an HBPC team will be identified from a combination of the HBPC Masterfile and DSS outpatient records of HBPC visits.

Since interventions will not be randomly assigned to CLC units or HBPC teams, we will need to control for selection bias, We will use propensity matching techniques to identify 1-3 matching CLC units or HBPC teams, with replacement from all CLCs and HBPC teams nationally. To estimate propensity scores we will develop a logistic regression model at the CLC unit level modeling the likelihood of a CLC unit to be included in our intervention. We will control for concurrent summary information at the unit level of location (urban/rural), associated VAMC (on-site with acute hospital, on-site without acute hospital, off-site), mix of short-stay, long-stay and hospice residents, number of beds and case-mix acuity measures aggregated from MDS assessments. To control for potential underlying trends in CLC unit context we will also test a subset of the covariates measured up to six prior quarters. The GEC DAC already develops many of these descriptive CLC indicators on a quarterly basis and will post them to the CLC Dashboard that is under development. We will use k-nearest neighbor with caliper propensity score matching with replacement to choose the three closest controls whose logit propensity score falls within one-fifth of the standard deviation of the mean logit propensity score for each CLC unit in the interventions group [40]. The same methods will be applied to aggregation of data over Veterans by HBPC team. The HBPC teams will be matched by rural or urban location, number of providers, average number of Veterans enrolled in the team and average length-of-stay in HBPC during the last year. All analyses will be conducted in Stata.

Our primary outcome, to measure progress on our overall goal, is the proportion of newly admitted veterans who have GoC conversations documented using the LST template within 7 days of admission to the CLC or within the second visit by HBPC staff. We will extract data for this outcome from VA’s Corporate Data Warehouse (CDW) Health Factors. The CDW is a national data resource, and the primary source of clinical and administrative data in the VA. While this primary outcome is essential to monitor progress for our impact goal, other outcomes are also important, noted in Table 1.

We will construct data files in which we assess trends in documentation and other outcomes by unit, CLC, HBPC team and program, facility, and regional network, at regular intervals. Ideally, this will occur every 2 weeks to permit segmented regression analysis using data beginning in the third year of implementation for summative evaluation. An important note is that the trended data will be used in both the feedback reports and in summative evaluation. Power for segmented regression analysis does not rely on the number of individuals contributing to each time point (either at the level of individual veteran or at the level of unit/team), but instead is dependent on the number of time points, both before and after the intervention begins [41]. We will construct this metric for the entire VA to permit comparison among participating facilities and others not participating in this program.

The primary outcome does not require adjustment because the requirement for documentation is the same regardless of individual veteran factors. However, we will use multivariable, hierarchical regression at key time points in each intervention to assess the impact of implementation and organizational factors. The unit of analysis will be both at the individual level (e.g., receipt of a palliative care consult, co-morbidities, physical and cognitive functioning) and at the unit or facility level (ORCA scales, barrier assessment, feedback uptake scores), which we list as covariates in Table 1.

## Discussion

### Limitations and challenges

We acknowledge that while the proposed projects cover many CLCs and HBPC programs, they are not fully representative of the full range of regional networks and facilities across the VA, and in some important areas that have a dense veteran population. One challenge already encountered is the fact that the updated Handbook will not be released to the field until after October 1, 2016, a year after the planned start of the project. We have initiated planned implementation activities with the four demonstration sites that have already installed and are using the LST template, and will continue with the projects described in this manuscript as soon as the LST initiative is released.

### Summary

This program will support implementation of a complex intervention to support veteran preferences for end-of-life care and relating to life-sustaining treatments. It is embedded in a large, national healthcare system providing care to over 6 million veterans in the USA, and will offer new insights into program implementation in large, complex systems in contexts that are not often studied in implementation science, namely post-acute and long-term care, and home-based care. The work conducted in this project is part of the VA’s quality improvement effort, and as such demonstrates VA’s capacity as a learning healthcare system.

## Additional files

Additional file 1: Individual project descriptions for: implementing goals of care conversations with veterans in VA long-term care setting: a mixed methods protocol. (DOCX 35 kb)

Additional file 2: Detailed implementation strategies for implementing goals of care conversations with veterans in VA LTC settings: a mixed methods protocol. (DOCX 22 kb)

Additional file 3: Issues to consider when QUERI-funded projects are considered quality improvement (5/15/15). (PDF 211 kb)

## Abbreviations

ACC: Artifacts of Culture Change
CFIR: Consolidated Framework for Implementation Research
CLC: Community Living Center; formerly “Nursing Home Care Unit”; units or facilities owned and operated by the VA
GEC: Geriatrics and Extended Care
GEC DAC: Geriatrics and Extended Care Data and Analyses Center
GoC: Goals of care
HBPC: Home-Based Primary Care
ICU: Intensive care unit
LST: Life-sustaining treatment
MDS: Minimum Data Set; comprehensive, standardized, federally mandated assessment tool that must be completed for every nursing home patient at admission and at regular intervals
ORCA: Organizational Readiness Change Assessment
PHQ-9: Patient Health Questionnaire: a widely used standardized questionnaire to assess the presence of depression
PROMISE: Performance Reporting and Outcomes Measurement to Improve the Standard of care at End-of-life
QI: Quality improvement
TDF: Theoretical Domains Framework
VA: Veterans Health Administration; part of the Department of Veterans Affairs
