# Correction to: Implementing goals of care conversations with veterans in VA long-term care setting: a mixed methods protocol

**DOI:** 10.1186/s13012-018-0724-y

**Published:** 2018-02-09

**Authors:** Anne E. Sales, Mary Ersek, Orna K. Intrator, Cari Levy, Joan G. Carpenter, Robert Hogikyan, Helen C. Kales, Zach Landis-Lewis, Tobie Olsan, Susan C. Miller, Marcos Montagnini, Vyjeyanthi S. Periyakoil, Sheri Reder

**Affiliations:** 1 Center for Clinical Management Research, VA Ann Arbor Healthcare System, Ann Arbor, MI USA; 2 University of Michigan Medical School, 300 N. Ingalls Street, Room 1161-I, Ann Arbor, MI 48109-5423 USA; 3 Corporal Michael J. Crescenz VAMC, Philadelphia, PA USA; 4 School of Nursing, University of Pennsylvania, Philadelphia, PA USA; 5 Canandaigua VAMC, Canandaigua, NY USA; 6 University of Rochester Medical Center, Rochester, NY USA; 7 Eastern Colorado Health Care System, Denver, CO USA; 8 School of Medicine, University of Colorado Anschutz Campus, Denver, CO USA; 9 Brown University School of Public Health, Providence, RI USA; 10 VA Palo Alto Health Care System, Palo Alto, CA USA; 11 Stanford University School of Medicine, Stanford University, Palo Alto, CA USA; 12 Puget Sound Health Care System, Seattle, WA USA

## Correction

The authors would like to correct errors in the original article [1] that may have lead readers to misinterpret the scope, evidence base and target population of VHA Handbook 1004.03 “Life-Sustaining Treatment (LST) Decisions: Eliciting, Documenting, and Honoring Patients’ Values, Goals, and Preferences”.

The original article [1] indicated that the target population for the practice changes described in the policy was *all Veterans*. In fact, the primary aim of the policy initiative is to ensure that the goals, values and life-sustaining treatment decisions of *Veterans at high risk for a life-threatening event within the next 1–2 years*, will be proactively elicited, documented, and then honored in the delivery of their care.

The original article [1] stated that goals of care conversations (GoCC) would be conducted with Veterans and their family members. Veterans with decision-making capacity will determine how or if they wish to have their significant others engaged in GoCC and life-sustaining treatment decisions. GoCC and subsequent life-sustaining treatment decisions for Veterans who lack capacity will occur with their duly authorized surrogate or health care agent in accordance with their advance directive. If a Veteran lacks capacity and does not have a surrogate, the policy describes a multidisciplinary process for decision making.

The original article did not note that all components of the VHA policy initiative were extensively field tested by the VA National Center for Ethics in Health Care at 4 VA medical centers that served as demonstration sites over a two year period. To date, over 7000 seriously ill Veterans have had their GoCC proactively elicited and documented on a standardized progress note template and order set located in the electronic health record.
