# Supplementary material for: Implementing goals of care conversations with veterans in VA long-term care setting: a mixed methods protocol
Source: Implement Sci. 2016 Sep 29;11:132. doi: 10.1186/s13012-016-0497-0 (PMC5041212; doi:10.1186/s13012-016-0497-0)
Supplement: Supplementary file 1 — Individual project descriptions for: implementing goals of care conversations with veterans in VA long-term care setting: a mixed methods protocol. (DOCX 35 kb) [file 13012_2016_497_MOESM1_ESM.docx]

**Individual Project Descriptions for: *Implementing Goals of Care Conversations with Veterans in VA Long Term Care Setting: A mixed methods protocol***

**Project 1: Implementing Goals of Care (GoC) conversations in Community Living Centers (CLC)s**

*Specific Aims*

While CLC staff currently strive to discuss GoC and preferences for life sustaining treatment with residents, these discussions and decisions are not always conducted and documented in a timely standardized fashion, leaving room for treatment against patients’ wishes. The updated guidance in Handbook 1004.03 being promulgated by the Veterans’ Health Administrations’ (VHA) National Center for Ethics in Health Care addresses these gaps. Specifically, the guidance requires that Veterans admitted to CLCs have GoC conversations completed and documented within 7 days of admission. It also requires that these conversations and their outcomes are documented in a new standard template which will be installed in a consistent, prominent place within the Computerized Patient Record System (CPRS) for easy access. With VHA’s Office of Geriatrics and Extended Care (GEC) policy to focus CLC care on short stays for hospice and post-acute care, an increasing number of Veterans (currently about 35%) spend an average of 26/40 days in the CLC (post-acute care/hospice).. As a result, there is a need for speedy completion of the GoC conversations, coupled with the need to elicit and plan for services related to Veteran preferences for post-discharge care, puts considerable stress on care teams to initiate and facilitate these conversations rapidly and effectively.

In this project, we aim to support implementation of the updated guidance in Handbook 1004.03 in Veterans Integrated Service Network (VISN) 11 (Michigan, Indiana, and one facility in eastern Illinois) CLCs. We will do this through the following specific aims:

1. Assess context, including current practices and resources available, throughout CLC units in VISN 11;
2. Assess barriers and facilitators to implementing these new practices using the Consolidated Framework for Implementation Research (CFIR) and Theoretical Domains Framework (TDF) to guide our assessment;
3. Using audit with feedback coupled with action planning derived from learning collaboratives, support ongoing implementation of GoC conversations, documentation, and enactment of GoC into care plans for individual Veterans.

*Rationale*

GoC conversations are widely accepted as critical to ensuring that patient preferences guide care decisions. In particular, understanding the treatment preferences of patients with advanced, life-limiting illness around life sustaining treatment is essential to ensuring that health care services are concordant with these preferences. Given the imperative to provide Veteran-centric care and enhance communication across care settings, it is critical to make sure that uniform documentation of treatment preferences is accessible regardless of care setting. These principles are central to the Physician Orders for Life-Sustaining Treatment (POLST) paradigm, which is an approach to advance care planning based on shared decision-making using GoC discussions among healthcare providers, patients, and their surrogates. Studies have shown that POLST improves congruence between patients’ preferences for care and the medical treatments that patients receive and is associated with lower use of aggressive, life-sustaining therapies[1].

Veterans Health Administration (VHA) Handbook 1004.03 seeks to implement a POLST-like approach in the VHA. It provides guidance for conducting GoC conversations, and includes a documentation template (the Life Sustaining Treatment Progress Note Template) that encodes Veterans’ preferences in a structured format in the VA electronic health record. The guidance focuses on Veterans with serious life-limiting medical conditions, and includes Veterans who receive care in a CLC. As strongly recommended in Handbook 1004.03, all Veterans should have a GoC conversation conducted and documented within 7 days of CLC admission. In this project, we focus on initial implementation of the guidance provided by the Handbook 1004.3, followed in the second phase in which we expand the focus to include an evaluation of the extent to which preferences documented through the Life Sustaining Treatment Progress Note Template (LST Template) are incorporated into individualized care plans for each Veteran. We will initially focus on Veterans newly admitted to CLC (as described in Handbook 1004.03) but will also work with CLC staff to ensure that all residents have documented GoC conversations, and that their care plans reflect their preferences.

*Procedures*

We will conduct the project as described in Additional File 2, which details the Implementation approaches of the Implementation and Data Core (IDC). We focus Project 1 on piloting feedback reports and learning collaboratives to ensure that we are able to get staff feedback as we develop and deploy these strategies.

*Proposed implementation/ improvement strategy*

Context assessment: We will begin with baseline context assessment in all CLCs in VISN 11, using the Organizational Readiness to Change Assessment (ORCA) Evidence and Context Assessments at baseline. These instruments consist of 34 items and can be administered online or in person. We will ask all CLC staff to complete these two ORCA instruments using web-based survey administration. In addition, we will have data from the Artifacts of Culture Change Tool to understand baseline practice, described earlier in the IDC section.

Barrier assessment: We will use the Consolidated Framework for Implementation Research (CFIR) and the Theoretical Domains Framework (TDF) to assess barriers to implementation of the Handbook 1004.03 guidance, including completion of the LST Template. We will conduct the barrier assessments through interviews, both in person and by telephone. We will use questions that have been developed for previous work from the CFIR Wiki[2], and from published reports using the TDF[3].

Audit with Feedback: We will use the audit with feedback strategy described in the description of the IDC. Initially, feedback reports will include at least three key indicators from the LST Template: proportion of eligible Veterans with Template completion within 7 days of admission; proportion of completed Templates in which the Veteran indicated preferences for full life sustaining treatments vs. less than full life sustaining treatments; and the proportion of completed templates in which the Veteran or surrogate expressed an understanding of his/her medical condition consistent with the medical facts. We will focus on these indicators during Phase 1 of the feedback intervention (Months 2-6).

In the second phase of the feedback intervention (Months 7-12), we will include additional indicators that reflect further depth in GoC conversations. Specifically, we will focus on the section of the Template which asks “What are the patient’s goals of care?” Veterans or their surrogates can select one or more of these goals. We will present these data to staff to help them individualize resident care plans based on the goals that were chosen.

Feedback reports will be delivered to unit staff and leaders monthly. We will download data from the VA Corporate Data Warehouse every week to populate the monthly feedback reports. In Phase 1 (first two quarters), we will deliver reports via email directly to individual staff members. In Phase 2 (second two quarters), we will randomly assign CLC units to receive feedback reports either directly through email to individuals, or through supervisory staff (attending physicians, nursing unit managers) who will receive coaching via email about ways to distribute and discuss feedback reports with unit staff along with the feedback reports. This will allow us to test these two distribution approaches and design elements for feedback interventions that have not as yet received much study.

Learning Collaborative : Shortly after initiating the Phase 1 feedback reports, we will recruit interdisciplinary teams consisting of representatives from direct care staff (including Certified Nursing Assistants (CNAs), licensed nursing staff , allied health professionals) and medical teams to participate in a Learning Collaborative. We will initiate this phase at the John D. Dingell VA Medical Center in Detroit, which has four CLC units with different services (short stay, long term care, dementia care) across the four units.

We will launch the learning collaborative with an in-person kick-off meeting, involving teams from all four units. We will present data from the feedback reports, blinded except for teams’ own unit. We will attempt to elicit positive steps to addressing issues or concerns with implementing the new documentation, ensuring that the team from the unit with the highest performance can share their practices and solutions with other teams. We will support the teams’ work by facilitating action planning through guided use of quality improvement tools, including a brief and focused root cause analysis, developing Specific-Measurable-Attainable-Realistic-Timely (SMART) goals, and facilitating team production of their own weekly action plans. We will follow up with each team every other week to assess their progress, and incorporate any new extractable data elements in the next cycle of feedback reports. Learning collaboratives will meet quarterly, incorporating the remaining CLC units in VISN 11 during the second quarterly meeting. We will meet face to face during the course of this project. The implementation elements we will modify and test include degree of project staff involvement in developing and monitoring action plans. As units improve performance, we will decrease the amount of staff monitoring of action plan progress. In units with little to no performance improvement, we will work more closely, conduct interviews with the team members, and assess team composition as well as team ability to enact action plans. This will become more critical in Phase 2, when the indicators reflect more complex processes (going beyond documenting conversations to the nature of the goals elicited). We will use the concurrent analysis of process evaluation data to guide tool development to provide additional support to lower performing units.

*Study design and methods*

Design: We will use before and after analyses with comparison groups to analyze both process and outcome data. While we are focused on VISN 11 CLCs for this project, we will conduct comparison analyses with matched CLC units from the other VISNs to compare with secular trend in completion of the LST template, our primary outcome for this project. In particular, we will match on proportion of short-stay residents, a group of particular interest and concern to GEC, who are often discharged to community, but may be quite frequently rehospitalized.

Settings: The boundaries of the VA regional networks were revised in October 2015, as this program was funded. We will work collaboratively with regional leadership to engage CLC leadership and staff throughout the area currently comprising VISN 11: Michigan, Indiana, and if appropriate, Ohio. We will include all CLCs within this area.

Data collection: We describe key data elements for this project in Table 1 in the main text. Details for each data element are described in more detail in the IDC section of the main text.

*Key outcomes, covariates, and analyses*

We will assess trends in the major outcome variable (proportion of eligible Veterans with complete LST documentation within 7 days of admission to CLC) over the period of the intervention. We cannot use an interrupted time series approach because it requires pre-intervention and post-intervention data in regular time intervals, and the timing of the Handbook release will make it impossible to collect pre-intervention data. Instead, for this project, we will use change in Bereaved Family Survey (BFS) scores for each CLC unit over the pre- and post- intervention period, focusing on response to the overall quality of care question of the survey (see description of the BFS in the main text). Scores are not available at the unit level, so this primary analysis will be at the full CLC level. We will have comparison scores for matched units throughout VHA using a 2:1 match based on facility size, proportion of short-stay residents, and rurality. We will conduct a difference-in-difference analysis at the facility level, differencing BFS scores from the beginning of the intervention to the end.

For secondary outcomes including the endorsement of full or less than full LST and proportion of Veterans expressing understanding of medical facts, we will also use multivariable regression at the unit level, controlling for unit culture using scales from both the Artifacts of Culture Change Tool and the VA All Employee Survey, administered to all VA employees annually. We will match intervened units with units from other VISNs on size and resident type, enabling a difference in difference design.

Using process evaluation data from the barrier assessment using CFIR and TDF we will use framework coding to analyze these data. We will also have data from the Feedback Report Uptake scale which we can use as a covariate in secondary analyses to understand the factors associated with changes in secondary outcomes, and data about completion of Action Plans from the learning collaboratives, which will also inform our analysis of the uptake and utility of the implementation strategies. We will be able to evaluate the differences in report uptake between units receiving different modes of feedback delivery in Phase 2, and differences in action planning completion as the learning collaboratives evolve.

*Impact*

We expect to see improvements in BFS scores over the period of the intervention, at the facility level, that are not explained by change based on the matched comparisons (secular trend). We also expect to see improvements over the period at the unit level in implementation of the core metric for the Impact Goal (i.e. proportion of Veterans with documented GoC conversations) that exceed the change in comparison units.

*Partnerships/Management*

The primary partner for this project is the VISN 11 Geriatrics and Extended Care (GEC) Committee. We will be working under their authorization to complete this quality improvement project, and will participate in their monthly teleconferences to report on the progress of the project. We will ensure that CLC Directors (members of the VISN 11 GEC committee) are fully apprised of our plans as we initiate this project, and ensure that we have their approval and staff knowledge of the project as we go forward. We will visit each CLC and meet with leadership and staff to explain the project, answer questions, and ensure open lines of communication as we proceed. In addition to our primary reporting to the VISN 11 GEC Committee, we will also report to our primary national program partners, the National Center for Ethics in Health Care, and the GEC Program Office. We will present our progress and findings at quarterly steering committee meetings, and as requested in briefings to both groups, as well as to the Office of Nursing Services.

**Project 3: Implementing Goals of Care Conversations in Home Based Primary Care (HBPC)**

**Specific Aims**

Updated guidance in the Life Sustaining Treatment Initiative (Handbook 1004.03) includes a new requirement that Veterans admitted to HBPC have Goals of Care Conversations (GoC conversations) completed and documented at the first or second visit with a VA HBPC practitioner. Establishing and documenting goals of care is consistent with key features of the HBPC program including: meeting the needs and preferences of Veterans and family throughout the course of chronic disease, often through end of life and allowing the Veterans the option of dying at home rather than an institution[4]. While completion of advance care plans in some VA HBPC programs exceed 95%[5, 6], the extent to which these Veterans with multiple chronic illnesses understand the benefits and burdens of life-sustaining treatments (LST) is not clear given that 69% of Veterans in HBPC indicated they preferred full resuscitative efforts in the event of cardiac arrest. Portability of documented care preferences is critically important in this cohort of vulnerable home-bound Veterans who frequently use emergency room and hospital services in VA and non-VA facilities[5, 7].

In this project, we aim to support implementation of GoC conversations and documentation consistent with the updated guidance in Handbook 1004.03 with Veterans and their families in HBPC programs in VISN 19. We will do this through the following specific aims:

1. Assess context, including current practices and resources available to conduct GoC conversations, throughout HBPC teams in VISN 19.
2. Assess barriers and facilitators to implementing these new practices using the Consolidated Framework for Implementation Research (CFIR) and Theoretical Domains Framework (TDF) as guiding frameworks.
3. Implement the new processes and procedures using information gathered in Aim 2 to minimize barriers and maximize facilitators.
4. Use audit with feedback coupled with action planning derived from learning collaboratives, support ongoing implementation of GoC conversations, documentation, and enactment of GoC into care plans for individual Veterans.

**Rationale**

National implementation of the Life Sustaining Treatment Initiative provides an opportunity to study implementation of this initiative in one of the target populations, Veterans enrolled in the HBPC program. HBPC enrollees are among the sickest and frailest of the aging Veteran population with, on average, six chronic conditions and 15 medications[8]. They receive an average of 3.1 home visits per month and typically remain in the program six to seven months[9]. Due to the high burden of illness in this population, frequent contact with care providers and high rates of advance directive documentation reported in prior studies[8, 9], evaluation of the LST implementation in this population is likely to provide important information about successful documentation strategies in chronically-ill home care populations. In addition, the previously raised concerns regarding Veterans’ understanding of the implications of selected treatment preferences, can be examined and can provide guidance on communication and educational approaches that lead to well-informed decisions. As of January 2016, there were 345 HBPC individually reporting teams operating in 138 programs nationally and an additional 50 teams which do not report individually. All VISN 19 teams will be required to implement the LST Initiative. We anticipate the close working relationship we have with our operational partners and with VISN 19 leadership will facilitate dissemination of the knowledge gained in this quality improvement project nationally to enhance implementation effectiveness.

**Procedures**

The following procedures will guide this project:

**Proposed implementation/improvement strategy**

Context Assessment (AIM 1): We will perform a context assessment of all HBPC programs in VISN 19 (Colorado, Montana and Oklahoma) (N=6). As described in Project 1 and in the IDC description, we will use the Organizational Readiness to Change Assessment Evidence and Context Assessments at baseline.

Barrier Assessment (AIM 2): Consistent with Project 1, we will use the CFIR and TDF to assess barriers to implementation of the Handbook guidance, including completion of the LST Template (See Implementation core for details) using both in-person and telephone interviews.

Audit with feedback (AIMs 3 & 4): As outlined in the Implementation Core section, the new processes and procedures developed in response to the knowledge gained about barriers and facilitators in AIM 2 will inform reports designed to provide actionable data to the teams (AIM 3). During Phase 1 of the feedback intervention, these reports will consist of: (1) proportion of eligible Veterans with LST Template completion within the 1^st^ or 2^nd^ visit; (2) proportion of completed LST Templates in which the Veteran indicated preferences for full life sustaining treatments vs. less than full life sustaining treatments; and (3) proportion of completed LST Templates in which the Veteran or surrogate expressed an understanding of his/her medical condition consistent with the medical facts.

Modeled after Project 1, Phase 2 of the feedback intervention (AIM 4) will include additional indicators to assess the content of goals of care conversations. Specifically, we will focus on the section of the LST Template which asks ***“What are the Veteran’s goals of care?”*** The question is asked of a family member or informal caregiver if the Veteran is unable to respond. The coded responses include: (1) describing the patient’s goals in their own words; (2) an open ended option “to be cured of…”; (3) to prolong life; (4) to improve or maintain function, independence, or quality of life; (5) to be comfortable; (6) to obtain support for family/caregiver; and (7) to achieve life goals, using an open ended response. We will use the responses to this section to provide feedback to staff on the proportion of Veterans with completed LST Templates who endorse each of these options and check for concordance between their understanding of preferences and documented preferences, as Veterans or their surrogates can select more than one goal.

In both feedback phases, reports will be delivered to the HBPC teams every month. We will download data from the Corporate Data Warehouse (CDW) monthly to populate the monthly feedback reports. In Phase 1 (first two quarters), we will deliver reports via email directly to individual staff members. In Phase 2 (second two quarters), we will randomly assign HBPC teams in both VISN 19 and 2 to receive feedback reports either directly through email to individuals, or through supervisory staff (attending physicians, nursing managers) who will receive coaching via email about ways to distribute and discuss feedback reports with HBPC staff along with the feedback reports. This will allow us to test these two distribution approaches and augment the knowledge gained in Project 1 with another care setting and team structure.

Learning Collaboratives – Learning collaborative teams will consist of HBPC clinicians from several disciplines (physicians, nurses, nurse practitioners, social workers, recreational therapists, dieticians) from HBPC interdisciplinary teams. A kick-off meeting will be attended by the Denver HBPC team members in person in Denver; the other kick-off meetings will be hosted via video-based communication for HBPC team members located in other cities. We will present data from the feedback reports across all six of the HBPC teams in VISN 19, blinded except for teams’ own location. Action planning will then be facilitated through guided use of quality improvement tools as described in Project 1. Learning Collaboratives will meet quarterly as is the standard of practice. The degree of project staff involvement in developing and monitoring action plans will be adapted based on team performance with greater input to teams that appear to have greater challenges. As the learning collaboratives evolve we will also introduce and reinforce principles of shared decision making.

**Study design and methods**

Design: We are focused on VISN 19 HBPCs in this project, but also will be extracting data on HBPC teams in VISNs 2, 4 and 11. Using these additional data, we will conduct comparison analyses with matched HBPC teams from the other VISNs to assess for trends in completion of the LST template. Matching will occur on rural vs. urban location and staffing model (physician vs. nurse practitioner led). We will use information from a recently completed survey of programs conducted in November 2015-April 2016 under a separate project in which more detailed information about the process employed by the program were reported.

Data collection: Key data elements for this project in Table 1 (IDC). Details for each data element are described in more detail in the IDC section.

*Key outcomes, covariates, and analyses*

Key outcomes are described in the IDC section with some minor modifications. The primary outcome variable will be the completion of the LST Template within the first or second visit of a physician/ nurse practitioner to a newly admitted HBPC patient (in contrast to within 7 days of admission for CLC patients) as specified in the LST Handbook. Bereavement surveys are not currently sent to families of Veterans dying at home while on HBPC so variables from the Bereaved Family Survey will not be included in the Project 3 analysis unless these surveys are extended to the HBPC population over the course of the project. Admission assessments using “Form 3” (see appendix) conducted on all HBPC admissions contain some information on function and caregiver support that correspond to some extent with those in collected on the MDS 3.0 in CLCs. Other measures of healthcare utilization and burdensome transitions will be included as markers of quality of care.

We will use interrupted time-series/segmented regression analysis techniques within the context of matched HBPC teams and comparative effectiveness analysis as described in the IDC for both process and outcome data. To analyze our primary outcome, the monthly probabilities of completion of the LST template on newly admitted Veterans within the 1^st^ or 2^nd^ visit, we will compare outcomes in the first year of the program to those prior to Project 3 initiation, as our pre-intervention period. To analyze the secondary outcomes including proportion of Veterans endorsing full or less than full LST, and proportion of Veterans expressing understanding of medical facts, we will also use multivariable regression at the team level, controlling for program characteristics. We will be able to propensity match teams in VISN 19 that receive our implementation intervention with teams from the other VISNs in our program, matching on rurality, number of Veterans served, average length of stay in program, team size and acuity of conditions.

We will also have process evaluation data from the barrier assessment using CFIR and TDF; we will use framework coding and analysis to analyze these data. We will have data from the Feedback Report Uptake scale which we can use as a covariate in secondary analyses to understand the factors associated with changes in secondary outcomes, and data about completion of Action Plans from the Learning Collaboratives (a form of fidelity checking) which will also inform our analysis of the uptake and utility of the implementation strategies. We will be able to evaluate the differences in report uptake between teams receiving different modes of feedback delivery in Phase 2, and differences in action planning completion as the Learning Collaboratives evolve.

Phase 3 in Year 4 will be used for evaluation of data of process and summative data, and to continue monitoring for sustainment of practices. Dissemination of successful implementation strategies will also be performed in collaboration with our strategic partners.

*Impact*

With over 300 HBPC teams nationally and approximately 55,000 enrollees in the program annually, identification of successful GOC conversation implementation strategies will be essential to ensure the Life Sustaining Treatment Initiative (Handbook 1004.03) achieves the intended benefits for Veterans and family/caregivers. Because rates of advance care planning among Veterans in HBPC are already high, the key impact of Project 3 is expected to be in better understanding of the content and quality of the conversations and LST documentation. LST documentation and the proportion of Veterans expressing understanding of medical facts are expected to improve over the course of implementation, which is a highly desired result of this quality improvement (QI) effort to ultimately ensure alignment between Veterans’ preferences and enactment of GoC into care plans for individual Veterans. We also anticipate findings from this QI effort will provide the evidence base needed to rapidly spread effective practices in GoC conversations to HBPC programs where the implementation strategy has not been employed.

*Partnerships/Management*

The primary partner for this project is the VISN 19 GEC. We will be working under their authorization to complete this quality improvement project. We will provide regular progress reports at regularly scheduled VISN-level meetings and visit each HBPC team to meet with leadership and staff to explain the project, answer questions, and ensure open lines of communication as we proceed.

As with the other projects, primary reporting will be to VISN 19 GEC partners but also to our primary national program partners, the National Center for Ethics in Health Care, and the Geriatrics and Extended Care Program Office. These presentations will occur at quarterly steering committee meetings, and as requested in briefings to both groups, as well as to the Office of Nursing Services.

References

1. Bernacki RE, Block SD: **Communication about serious illness care goals: A review and synthesis of best practices.** *JAMA Internal Medicine* 2014, **174**(12):1994-2003.

2. Damschroder LJ, Aron DC, Keith RE, Kirsh SR, Alexander JA, Lowery JC: **Fostering implementation of health services research findings into practice: a consolidated framework for advancing implementation science.** *Implementation Science* 2009, **4**:50.

3. Francis JJ, O'Connor D, Curran J: **Theories of behaviour change synthesised into a set of theoretical groupings: introducing a thematic series on the theoretical domains framework.** *Implement Sci* 2012, **7**:35.

4. Beales JL, Edes T: **Veteran's Affairs Home Based Primary Care.** *Clin Geriatr Med* 2009, **25**(1):149-54, viii-ix.

5. Chang C, Jackson SS, Bullman TA, Cobbs EL: **Impact of a home-based primary care program in an urban Veterans Affairs medical center.** *J Am Med Dir Assoc* 2009, **10**(2):133-137.

6. North L, Kehm L, Bent K, Hartman T: **Can home-based primary care: cut costs?** *Nurse Pract* 2008, **33**(7):39-44.

7. De Jonge KE, Jamshed N, Gilden D, Kubisiak J, Bruce SR, Taler G: **Effects of home-based primary care on Medicare costs in high-risk elders.** *J Am Geriatr Soc* 2014, **62**(10):1825-1831.

8. Tervo-Heikkinen T, Kiviniemi V, Partanen P, VehvilÄinen-Julkunen K: **Nurse staffing levels and nursing outcomes: A Bayesian analysis of Finnish-registered nurse survey data.** *J Nurs Manag* 2009, **17**(8):986-993.

9. Cepeda-Valery B, Pressman GS, Figueredo VM, Romero-Corral A: **Impact of obesity on total and cardiovascular mortality-fat or fiction?** *Nature Reviews Cardiology* 2011, **8**(4):233-237.
