# Supplementary material for: Implementing goals of care conversations with veterans in VA long-term care setting: a mixed methods protocol
Source: Implement Sci. 2016 Sep 29;11:132. doi: 10.1186/s13012-016-0497-0 (PMC5041212; doi:10.1186/s13012-016-0497-0)
Supplement: Supplementary file 2 — Detailed implementation strategies for implementing goals of care conversations with veterans in VA LTC settings: a mixed methods protocol. (DOCX 22 kb) [file 13012_2016_497_MOESM2_ESM.docx]

**Additional File 2: Detailed Implementation Strategies for *Implementing Goals of Care Conversations with Veterans in VA LTC Settings: A Mixed Methods Protocol***

*Audit with feedback as an implementation strategy*

Audit with feedback has been extensively studied as an approach to modifying behavior among specific groups of providers[1]. Audit with feedback involves aggregating clinical or other performance data, both over time and, in the case of unit or team feedback, over individual performance, and providing the aggregated data summary to individual practitioners, teams, or healthcare organizations[1]. It has been shown to have a positive but modest absolute effect of increasing the likelihood of achieving a desired behavior change of about 4%, at the median[1]. Despite a strong body of literature, little is known about how to optimize effectiveness of feedback interventions. Key design features of audit with feedback interventions include specifying the group being targeted by the feedback intervention; format of the report (including whether verbal or written; length and complexity; display, such as use of text vs. graphics among other features); mode of delivery; frequency of delivery (how often reports are delivered within a specific period of time, such as a year); optimal duration of intervention; and the framing of the report (whether the report is intended to draw attention to positive or negative aspects of past performance)[2, 3].

Related to this latter point, van Dijk and Kluger[4, 5] have proposed that how someone responds to a feedback report can vary depending on the context in which the report is received and the perceived positive or negative sign of the report. For example, if the context is perceived as punitive and psychologically unsafe, a positive report can result in the recipient decreasing effort as part of a relaxation response, reacting in the opposite manner from what was intended. Similarly, in a context in which the recipient feels safe and is not concerned that punitive action will be taken, a negative report can result in feelings of discouragement, leading to task avoidance or decreased behavior change.

Several key features of effective audit with feedback have been described, derived either through theory or empirical evidence, in systematic reviews with meta-analysis. These include timeliness, actionability, validity, and non-punitive approaches[6, 7]. Some of these features are readily modifiable, while others are not, even though they may appear modifiable or under the control of the feedback provider. Timeliness is particularly problematic, as it is not always possible to provide aggregated data within a period considered sufficiently timely by the recipient. Frequency of feedback reports is usually tightly coupled to the velocity of data flow. When data are refreshed at relatively infrequent intervals, or the volume of cases specific to the feedback topic is not large, it can be difficult to produce sufficiently timely data, or to meet the expectations of feedback report recipients.

Actionability is linked to timeliness. Recipients often consider “old” data to be non-actionable, and often cite this as a reason for not using feedback reports to change behavior. While this is an important area, the literature on this issue is somewhat sparse. In our review of the issue of actionability and timeliness, a key area of disconnect is evident in the literature. Studies of feedback interventions fall into two large groups. The first group is experimental data, largely done as psychology studies, often with university student subjects. Experiments are rapid, short approaches to assigning a task to the subject (which can often be completed within a very short timeframe such as seconds or minutes), assessing their performance, and feeding it back to the subject. These lab-based experiments have provided valuable information about how humans react to performance feedback, but bear little relationship to real-world feedback in the context of work settings. The other large group of studies are those done in work settings, with performance feedback to individuals or groups that uses data about complex, time-consuming tasks which often require much longer intervals to complete. These are included in the systematic reviews and meta-analyses of audit with feedback interventions in health care[1]; intervals included in these studies ranged from a few weeks to more than a year. These two different realities constitute a serious area of disconnect between the psychological theories of feedback interventions and the pragmatic realities of delivering feedback in health care settings using clinically relevant, transactional data. The speed at which data accumulate to a level where they can be aggregated, for example for more than 10-20 individuals, which can also be conceptualized as the velocity of the data, varies widely. Data are often quite sparse for a given clinical condition and setting, a problem pointed out by Hofer et al.[8] for diabetes care. As a result, deriving statistically valid numbers for each time point at which data are aggregated and included in a feedback report can be a lengthy and complex process.

Several mechanisms, based on psychological and psycho-social theory, have been proposed to enhance the effectiveness of feedback interventions. Coupling feedback reports with action plans is one approach that has been strongly recommended, with some evidence to support it. In particular, goal setting with feedback, which can be derived through action plans, is strongly theory-based[9].

*Learning collaboratives and action planning*

Learning collaboratives are defined as multidisciplinary teams coming together from different divisions internal to a single organization or from across multiple organizations to work in a structured way to improve quality of care. The structure is typically a kick-off meeting, followed by up to 18 months of periodic meetings (often quarterly), with check-in by collaborative facilitators[10]. In the classic Institute for Healthcare Improvement approach, collaboratives meet monthly by phone and quarterly in person, with sharing of experiences and best practices to support learning and action by teams within their own organizations[11].

A relatively large literature describes approaches to designing and deploying learning collaboratives. There have been at least two systematic reviews of learning collaboratives and their effectiveness in promoting desired behavior change, as well as syntheses of lessons learned from learning collaboratives[12]. The literature shows that some elements of learning collaboratives are more effective, including a focus on data; team cohesion; organizational context; collaborative faculty or facilitators for the collaborative; creation of a change package; and use of quality improvement tools, such as Plan-Do-Study-Act cycles[10].

It is not clear how effective learning collaboratives have been in (i) supporting desired change, and (ii) sustaining or institutionalizing change once achieved. In some instances, the expected outcomes are relatively poorly specified, while in others, they are specific and clear. One important outcome of most learning collaboratives is sharing of approaches to resolving commonly encountered barriers. A component of overcoming barriers is anticipatory planning, which is strongly related to action planning, frequently described as an important approach to improving the effectiveness of feedback interventions.

Action planning is the use of systematic, structured approaches to planning activities designed to meet a specific goal. In the context of feedback interventions, action planning refers to planned, systematic approaches to responding to gaps in performance noted through feedback reports. Action planning is generally an important component of learning collaboratives, which routinely use data fed back for the purpose of generating actions to improve quality of care in specific ways. We will deploy learning collaboratives primarily for the purpose of supporting action planning in association with feedback reports.

*How we will develop and deploy feedback reports*

We will collect data retrospectively from existing records for all feedback reports. This creates both efficiencies and a more sustainable approach. Using project-specific data means that when the project ends, the data stream ends, and the reports can no longer be generated. VA has massive data resources, some of which are routinely used for feedback, and others which have not yet been tapped. Our primary outcome, by which we will measure progress on the impact goal of supporting implementation of goals of care conversations in Veteran Health Administration (VA) Long Term Services and Supports (LTSS), is the use of the standardized Life Sustaining Treatment Progress Note Template (LST Template), required as part of the implementation of Handbook 1004.03. Data are encoded from the LST Template into specific fields in the Health Factors table that exists as part of the electronic health record for all VA facilities, and can be linked to patient-specific, identified records. Health Factors fields can be extracted from data warehouses at either the Veteran, facility or regional level. We will work with the Office of Information Technology staff at each site to work out a strategy for extracting these fields. Free text can also be entered into the template, and we will work out strategies for extracting these data.

Other data sources will provide triangulation of the data. One of these is from the Minimum Data Set 3.0 (MDS 3.0), Section F: Preferences for Customary Routine and Activities, which consists of two sections for residents, dealing with daily routine preferences (choice of clothing, choice of bedtime) and activity preferences (having reading materials, getting fresh air in good weather), or one section which staff complete if the resident cannot. While these preferences are not the same as those focused on in the LST Template, there may be overlap, and we will assess overlap.

We will develop short, focused feedback reports related to completion of the LST Template. All reports will be constructed at the level of the Community Living Center (CLC) – i.e., VA Nursing Home unit, a geographic unit with specified rooms and common areas. The first data element will be the proportion of newly admitted Veterans with LST Template completed within 7 days of admission to the CLC. Another data element will be the completeness and personalization of the LST Template. Personalization will be assessed by comparing across newly admitted residents to assess whether the information in the template is personalized for a specific Veteran rather than being homogenous for all Veterans in a unit. A third element will be the proportion of Veterans for whom full LST are documented, versus those with less than full LSTs. Finally, we will include the proportion of Veterans or their families who express understanding of the Veteran’s medical condition consistent with the medical facts. This latter element reflects the degree to which the practitioner engaging in the goals of care conversation has been able to adequately communicate the health status and other aspects of the Veteran’s health needs. This is important because it provides an assessment of the adequacy of the discussion, and sheds light on the decisions being made about whether or not full life sustaining measures should be provided.

The reports will be graphical in nature, visualizing proportions of Veterans in each category for each element. The rates for each unit will be shown in comparison to other similar units in VISN 11, matching units on number of beds and resident type (e.g. short stay vs. long stay). There will be brief text in bullet form describing the sources of data, and as the intervention proceeds, whether or not the rate is higher or lower than in the previous time period. This is similar to feedback reports used in a previous study[13, 14] in Canadian nursing homes, in which the reports were widely read and understood by all types of staff in long term care units[14]. However, even within these parameters, the format can be varied slightly at each time period to assess the effect of different formats and design. Very little is known about optimal design of feedback reports, so this will be important new knowledge.

The frequency of the reports will depend on the rate of new admissions to a CLC unit. Assuming a 40-bed unit, and approximately 25% monthly turnover (not uncommon in a short-stay unit), we would expect to have approximately 10 new residents admitted each month. This would produce slightly more than two new residents per week on average. With this rate of new residents, it would be feasible to produce monthly reports as there will be sufficient numbers of new residents every month. If the turnover rate in the unit is higher, or if the unit is larger, it may be feasible to generate reports every two weeks. We will assess this as part of the baseline assessment for each CLC unit. Balancing between increasing the frequency of feedback reports and the rate at which the data are refreshed, or the velocity of the data, is a complex process. We will use the initial quarter in each new project to assess these characteristics and decide how frequently to deliver feedback.

An important question for any implementation intervention is whether or not the intervention has actually been received by the target recipients, i.e., the fidelity and dose of the intervention. This is especially important for feedback reports, which can only provide a mechanism for action if they are actually received, read, and at least minimally understood. In previous work, we developed a feedback uptake scale which is a parsimonious brief survey consisting of five questions that feedback recipients are asked to answer[14]. Our method of administering this brief survey will depend on how feedback reports are being delivered. If by hand, we will attach a paper version of the survey to the feedback report, and ask staff to complete it and drop it into a box in the unit. If electronic, we will follow up with participants within one week to request that they respond electronically to the survey.

The text used in the feedback reports is one element we will vary and experiment with. There are no clear standards for the tone, amount, or type of information in textual elements of feedback reports. In many studies, this is not described with enough detail to make any assessment of how text was used. In work being done using other kinds of messages to both patients and providers, messages tailored to characteristics of the individual receiving feedback have been shown to be effective[15]. Our approach will be to develop methods of tailoring to context, based on the context assessed in the unit, as well as the barriers and facilitators assessed during the baseline assessment period. We will develop a knowledge-base of these data, and methods to refine textual messages to fit the context. One of our experiments will be to test the effectiveness of these context-tailored messages against non-tailored messages, another area in which we do not have good information about optimal feedback report design.

We will deploy the feedback reports using different methods. Our initial approach, as we build rapport with staff and establish relationships, will be to hand deliver reports, as we did in previous work[14]. We will test this method against alternatives, such as email delivery, web-based delivery, delivery through supervisors, and delivery through learning collaboratives alone. Again, this is important information to help design optimal methods of delivering feedback. However, as we progress through the program, we will design more general approaches to feedback, such as dashboards. Dashboards are tools that are of primary benefit to managers rather than frontline clinicians, making them less appropriate for the specific, individual and team focused feedback reports we will focus on as a primary implementation approach in this program. Dashboards may be more useful in sustaining change, as they allow managers and systems personnel at higher levels of the organization to monitor whether gains are sustained, and decide whether booster efforts are needed to maintain performance. It is likely that in the routinized phase—that is once implementation has been successful—dashboards are important tools for system maintenance. Dr. Orna Intrator, PI of the Data component of the program’s Implementation and Data Center (IDC), has substantial experience using existing data to create dashboards. She will lead development of dashboards to monitor ongoing performance of elements related to the LST Handbook.

*How we will develop and deploy learning collaboratives*

We will initiate a learning collaborative in Project 1 in a single, multi-unit CLC. This will allow us to begin the learning collaborative using face-to-face methods, building relationships with staff and assessing team formation. We will follow the Institute for Healthcare Improvement Break-Through Collaborative model[11], with an initial sharing meeting in which the teams from at least two units meet together, share feedback reports with each other, and share approaches to plan for improvement in the performance metrics in their feedback reports. We will provide information and access to quality improvement tools and methods, and sample action plans for their use in developing unit-specific action plans.

Several tools and handbooks with varying degrees of sophistication and complexity have been developed to support quality improvement activities in health care settings. We will provide several different kinds of samples, and get feedback from staff about which appear most useful to them, including any that are already in use in the units. After we have conducted 3-4 feedback sessions with staff, we will standardize to the most commonly preferred handbook and toolkit, and use that as our standard, base support. We will continue to elicit feedback about the tools and handbook we use, and change if there is a shift in preference.

We will adhere to principles of well-designed learning collaboratives to the extent that these are known and agreed on. These include: identifying a shared problem/concern for improvement that is aligned with the strategic priorities for the organization; collaborative member involvement in defining the problem and measurable objectives for improvement; administrative leadership support to ensure sufficient resources and to eliminate barriers to group success; group members spending in-person time together; data sharing; a communication system to optimize the flow of information in all directions; experts in the field to develop knowledge of the group about the problem and of standards and performance targets; access to data and performance reports; training and ongoing coaching in the improvement method; and regular reporting sessions for sharing successes, problem-solving, and refining action plans[11].

References

1. Ivers N, Jamtvedt G, Flottorp S, Young JM, Odgaard-Jensen J, French SD, O'Brien MA, Johansen M, Grimshaw J, Oxman AD: **Audit and feedback: effects on professional practice and healthcare outcomes.** *Cochrane Database Syst Rev* 2012, **6**:CD000259.

2. Ivers NM, Sales A, Colquhoun H, Michie S, Foy R, Francis JJ, Grimshaw JM: **No more 'business as usual' with audit and feedback interventions: towards an agenda for a reinvigorated intervention.** *Implement Sci* 2014, **9**:14-5908-9-14.

3. Brehaut JC, Colquhoun HL, Eva KW, Carroll K, Sales A, Michie S, Ivers N, Grimshaw JM: **Practice Feedback Interventions: 15 Suggestions for Optimizing Effectiveness.** *Ann Intern Med* 2016, **164**(6):435-441.

4. Van-Dijk D, Kluger AN: **Feedback Sign Effect on Motivation: Is it Moderated by Regulatory Focus?** *Appl Psychol* 2004, **53**(1):113-135.

5. Kluger AN, Van Dijk D: **Feedback, the various tasks of the doctor, and the feedforward alternative.** *Med Educ* 2010, **44**(12):1166-1174.

6. Hysong SJ, Best RG, Pugh JA: **Audit and feedback and clinical practice guideline adherence: Making feedback actionable.** *Implement Sci* 2006, **1**:9.

7. Hysong SJ: **Meta-analysis: audit and feedback features impact effectiveness on care quality.** *Med Care* 2009, **47**(3):356-363.

8. Wilcoxen KM, Hesterman J, Orcutt KD, Hoppin J: **Intersectional innovation in biomarker development for patient-centric medicine.** *Personalized Medicine* 2011, **8**(4):469-481.

9. Foti ME, Geller J, Guy LS, Gunderson JG, Palmer BA, Smith LM: **Borderline personality disorder: Considerations for inclusion in the Massachusetts Parity List of "Biologically-based" disorders.** *Psychiatr Q* 2011, **82**(2):95-112.

10. Nembhard IM: **Learning and improving in quality improvement collaboratives: Which collaborative features do participants value most?: Quality and patient safety.** *Health Serv Res* 2009, **44**(2P1):359-378.

11. Syed IAA: **Glycated haemoglobin; past, present, and future are we ready for the change.** *Journal of the Pakistan Medical Association* 2011, **61**(4):383-388.

12. Schouten LMT, Hulscher MEJL, Van Everdingen JJE, Huijsman R, Grol RPTM: **Evidence for the impact of quality improvement collaboratives: Systematic review.** *BMJ* 2008, **336**(7659):1491-1494.

13. Sales AE, Schalm C, Baylon MA, Fraser KD: **Data for improvement and clinical excellence: Report of an interrupted time series trial of feedback in long-term care.** *Implementation Science* 2014, :161.

14. Sales AE, Fraser K, Baylon MAB, O'Rourke HM, Gao G, Bucknall T, Maisey S: **Understanding feedback report uptake: Process evaluation findings from a 13-month feedback intervention in long-term care settings.** *Implementation Science* 2015, **10**(1).

15. Hermanns M: **Culturally competent care for parkinson disease.** *Nurs Clin North Am* 2011, **46**(2):171-180.
