# Supplementary material for: Implementing goals of care conversations with veterans in VA long-term care setting: a mixed methods protocol
Source: Implement Sci. 2016 Sep 29;11:132. doi: 10.1186/s13012-016-0497-0 (PMC5041212; doi:10.1186/s13012-016-0497-0)
Supplement: Supplementary file 3 — Issues to consider when QUERI-funded projects are considered quality improvement (5/15/15). (PDF 211 kb) [file 13012_2016_497_MOESM3_ESM.pdf]

Issues to Consider When QUERI-Funded Projects are Considered Quality Improvement (5/15/15)

The VHA QUERI program was established in 1998 to more rapidly translate research into practice. Since its inception, many QUERI projects have involved the application of implementation science, which includes the systematic study of mechanisms to improve translation of research findings into practice. Because these projects are often investigator-initiated, involve collection of data not routinely available in clinical care, and may lead to generalizable knowledge, in many cases they have been required to undergo IRB review. At the same time, some QUERI projects involve extensive input or direction from VA operational partners and are not designed to contribute to generalizable knowledge but rather benefit a VA program or office. Moreover, because QUERI is funded by medical care special purpose funds, these projects are eligible to be considered as quality improvement (QI) and not research.

The following summary provides additional guidance to QUERI investigators who plan to conduct QI projects. This summary is largely based on VHA Handbook 1058.05 and based on further discussions with the VHA Central IRB (cIRB) and the VHA Office of Research Oversight regarding whether some QUERI-funded projects could be considered QI, and the implications for designating QI projects within QUERI and VHA. It is important to note that the boundary between research and QI can be difficult to discern, so this summary is a work in progress and will likely be revised based on ongoing discussions with cIRB and anticipated revisions to the Common Rule (e.g., see http://www.thehastingscenter.org/LearningHealthCareSystems/).

1. Determining whether a National (i.e., Partnered Evaluation Initiative) or local (i.e. facility or VISN level) QUERI project is considered Quality Improvement (QI) per VHA Handbook 1058.05

Since QUERI projects are funded by medical care and not research (ORD) funds, they are eligible to be considered Quality Improvement (QI) per the VHA Handbook 1058.05 and thus would not require IRB review.

a. For projects involving a **NATIONAL operations partner such as a Program or Leadership Office**, QI status must be documented via a letter from one of the VHA program officials authorized to provide documentation (see http://www.va.gov/ORO/oropubs.asp for the list). The letter should explain that the project meets the Handbook's criteria for QI, including: 1) the project is designed and implemented for **internal** VA purposes (i.e., findings are intended to be used by and within VA or by entities responsible for overseeing VA and not inform activities beyond VA i.e. not generalizable) , 2) the project is **not** designed to produce information that expands the knowledge base of a scientific discipline or field, and 3) the project does **not** involve collecting new data that are not needed for internal operations purposes but would otherwise produce information that expands the knowledge base.

b. For projects involving a **LOCAL facility or VISN**, each Facility or VISN should have their own standard operating procedures for handling QI projects, so a letter from a national program office of other designee is not required. Instead, the facility or VISN Director or his/her designee can provide a similar letter documenting QI status, or many facilities designate their IRB or R&D to provide documentation by email for projects considered QI (in both cases referencing guidelines from VHA Handbook 1058.05 is helpful). For these local projects, investigators are strongly encouraged to obtain documentation of QI status.

2. Issues to consider if program meets criteria for QI:

- Projects that use systematic methods, such as stepped wedge or clustered designs may still be considered QI, as long as the project is not designed to contribute to "generalizable knowledge" outside of VA per VHA Handbook 1058.05 (http://www.va.gov/vhapublications/ViewPublication.asp?pub_ID=2456)
- Datasets created for QI projects are not considered research if the data are being used for internal operations purposes only. Otherwise these datasets may need to comply with VHA research repository guidelines per VHA Handbook 1200.12: http://www1.va.gov/vhapublications/ViewPublication.asp?pub_ID=1851
- Many journals accept QI studies as long as documentation is provided indicating that the project is considered QI
- Documentation that the QI project is exempt from the Paperwork Reduction Act requirements will be needed from the QUERI Program Office if the project involves primary data collection from VA patients or participants outside the VA per OMB waiver guidelines (5 CFR Part 1320.3). Surveys or interviews that satisfy exemption involve 1) data collected re: routine clinical care/assessments/treatment designed to provide clinical examination and/or contribute to prevention of clinical disorders, and 2) are not considered duplicative. OMB waivers are not required for surveys or interviews of VA providers or managers.

3. Please send QI and OMB documentation (if applicable) to QUERI Program Office staff prior to funding. Contact QUERI Program Manager Linda McIvor (Linda.McIvor@va.gov) for QI letter and OMB waiver documentation templates**.**
